# Challenging diagnosis and treatment of rare paranasal sinus metastasis from thyroid cancer: a case report and literature review

**DOI:** 10.3389/fendo.2025.1550831

**Published:** 2025-05-05

**Authors:** Jee Hee Yoon, Ji Yong Park, A Ram Hong, Sung Sun Kim, Hee Kyung Kim, Ho-Cheol Kang

**Affiliations:** ^1^ Department of Internal Medicine, Chonnam University Medical School, Hwasun, Republic of Korea; ^2^ Division of Endocrinology and Metabolism, Chonnam National University Hwasun Hospital, Hwasun, Republic of Korea; ^3^ Department of Pathology, Chonnam University Medical School, Hwasun, Republic of Korea

**Keywords:** paranasal sinus, metastasis, thyroid cancer, follicular thyroid carcinoma, lenvatinib

## Abstract

**Background:**

Metastasis of thyroid carcinoma to the paranasal sinuses is extremely rare. Herein, a case of clinically occult thyroid cancer arising from a long-standing thyroid nodule that metastasized to the sphenoid sinus is presented, accompanied by a literature review.

**Case presentation:**

A 48-year-old woman visited to the otorhinolaryngology department with visual disturbance and partial nasal obstruction. Computed tomography imaging revealed a tumor in the right sphenoid sinus with adjacent bony destruction, suggestive of paranasal sinus cancer. Thyroid ultrasonography (US) was performed to identify the primary cancer, revealing a thyroid nodule previously diagnosed as a benign follicular nodule 11 years prior. Histopathological examination confirmed minimally invasive follicular thyroid carcinoma (FTC) and poorly differentiated thyroid carcinoma with sphenoid sinus metastasis. Lenvatinib therapy was initiated after total thyroidectomy and radioactive iodine (RAI) therapy, achieving stable disease for 29 months. The patient ultimately succumbed to newly developed brain metastasis with cerebral infarction, 31 months after the initial diagnosis. A literature review of 19 cases revealed that FTC was the most common type (68.5%), followed by papillary thyroid carcinoma (31.6%). Among the 12 cases with assessable clinical outcomes, three patients achieved remission, while nine had persistent disease, with four confirmed deaths.

**Conclusion:**

Diagnosis of paranasal sinus metastasis from thyroid cancer is often delayed due to its rarity and is commonly associated with widespread disseminated disease, resulting in a poor prognosis. Careful follow-up of large thyroid nodules and clinical suspicion of unusual metastasis is essential for early detection of malignancy and metastasis. Multidisciplinary collaboration and various treatment approaches can improve treatment efficacy.

## Introduction

Differentiated thyroid carcinoma (DTC), including papillary thyroid carcinoma (PTC) and follicular thyroid carcinoma (FTC), is generally indolent with a favorable prognosis (1, 2). However, up to 9% of DTC patients present with distant metastasis, depending on the study population and follow-up duration (3–5). The most common sites of distant metastasis are the lungs, bones, and brain, while metastasis to unusual sites is extremely rare (6). Most recurrent or persistent cases of DTC are detected in the neck; therefore, thyroid ultrasonography (US) is primarily used, with additional tests conducted for suspected distant metastasis, typically in the lungs and bones (2, 7). Due to the rarity of metastasis to unusual sites, diagnosis and treatment might be delayed. In cases where patients present with symptoms from unusual metastasis before a thyroid cancer diagnosis, diagnostic challenges might be greater than in patients already undergoing follow-up. Metastasis to the paranasal sinus is uncommon, with the most common primary cancers being from the kidney, urogenital, and breast (8). Thyroid cancer metastasis to the paranasal sinus is extremely rare, primarily reported in case studies, and presents unique diagnostic and therapeutic challenges.

Herein, a case of a patient presenting with a paranasal sinus tumor initially resembling paranasal sinus cancer, later diagnosed as metastasis from thyroid cancer. The patient received integrated, multidisciplinary care and was treated with a tyrosine kinase inhibitor. This report aims to describe the clinical features, diagnostic process, and treatment of paranasal sinus metastasis from thyroid cancer, along with a literature review.

## Case presentation

A 48-year-old female presented to the otorhinolaryngology department with a 3-month history of right-sided visual disturbance and partial nasal obstruction, along with episodes of nausea and vomiting over several weeks. She had a thyroid nodule diagnosed as a benign follicular nodule by fine needle aspiration (FNA) at a local hospital 11 years prior. A follow-up US 3 years later showed an increase in the size of the thyroid nodule, and repeat FNA was recommended, though the patient declined. She had no family history of thyroid disease or history of smoking or alcohol use. Physical examination revealed a protruding mass in the right superior ear region. A paranasal sinus computed tomography (CT) scan revealed a heterogeneously enhancing mass measuring approximately 3.8 × 3.6 × 3.3 cm in the right sphenoid sinus, with adjacent bony destruction in the cervical spine, suggestive of paranasal sinus cancer (**Supplementary Figure 1**). A right sphenoidotomy with biopsy was performed, and histology showed nodular follicular hyperplasia. Additional immunohistochemical staining indicated positivity for thyroid transcription factor-1 (TTF-1) and paired box gene 8 in the tumor cells. Thyroid US demonstrated multiple thyroid nodules and enlargement of the primary nodule, which exhibited incomplete rim calcification and an increase in size from 3.2 cm to 4.2 cm compared to the US conducted 8 years prior (**Supplementary Figure 2**). Cytology from repeat FNA indicated a follicular neoplasm. Endoscopic sinonasal surgery with sphenoidotomy and right orbital decompression was performed to remove the metastatic tumor and relieve symptoms, followed by total thyroidectomy. The histopathological analysis identified a 4.5 cm minimally invasive FTC and poorly DTC (PDTC) with lymphovascular invasion, central lymph node metastasis, and sphenoid sinus metastasis (T3aN1aM1) (**Supplementary Figure 3**).

A whole-body fluorodeoxyglucose -18F ( (18) F-FDG) positron emission tomography/CT scan revealed multiple bone metastases and palliative radiation therapy was administered to the sphenoid sinus, spine (cervical and lumbosacral), and left pelvic bone. A dose of 150 mCi of radioactive iodine (RAI)-131 was administered, with post-RAI imaging showing high (18) F-FDG uptake in lesions in the sphenoid and C3 and T12 vertebrae (**Supplementary Figure 4**). After prior treatments, it was decided to reassess additional RAI therapy for regions with minimal (18) F-FDG and iodine uptake. Additional palliative radiation therapy was administered to the T7 and T12 vertebrae, as well as the left tibia. Lenvatinib therapy was initiated at a dose of 24 mg 2 months after the initial diagnosis; however, due to uncontrolled proteinuria, hypertension, and diarrhea, the dose was reduced to 14 mg. Next-generation sequencing analysis identified a neuroblastoma RAS viral oncogene homolog mutation with a low tumor mutation burden. Local symptom management was provided by the otorhinolaryngology department for paranasal discomfort. Ophthalmology consultation was conducted for vertical diplopia and ptosis, and a facial nerve block was administered to control pain. After lenvatinib therapy initiation, paranasal sinus, chest CT, and spine MRI were performed at intervals of 3 to 6 months. If symptoms occurred, additional abdomen and brain imaging was also conducted. During the 29-month lenvatinib treatment, the disease remained stable. The serum thyroglobulin level, which had been measured above 5000 after surgery and palliative radiation therapy, decreased to 2040 after RAI therapy. It rapidly decreased after starting lenvatinib therapy and showed the lowest level at 97.8 ng/dL after one year of lenvatinib therapy (**Supplementary Figure 5**). However, 1 month after the last regular visit, the patient presented to the emergency department with altered mental status and subsequently died due to cerebral infarction in basal ganglia, frontal bone, and uncus, along with newly detected brain metastasis.

A review was conducted by searching for English-language papers in the PubMed, Medline, EMBASE, and Scopus databases. Search terms included ‘thyroid,’ ‘cancer,’ ‘malignancy,’ ‘thyroid cancer,’ ‘thyroid metastasis,’ and ‘metastasis,’ combined with ‘sphenoid,’ ‘ethmoid,’ ‘maxillary,’ ‘paranasal sinus,’ and ‘nasal cavity.’ Studies lacking full-text access, with uncertain diagnoses of thyroid cancer, or involving only facial bone metastasis were excluded. A literature review was conducted on 19 cases, including this case (**Table 1**). Synchronous metastasis was defined as a time interval of less than 6 months between the initial diagnosis of primary thyroid cancer and the diagnosis of paranasal sinus metastasis. Statistical analysis was performed using Statistical Package for the Social Sciences (SPSS) version 23.0 (SPSS Inc., Chicago, IL), with data expressed as medians (interquartile range) or n (%).

**Table 1:** Reported cases of paranasal sinus metastasis from thyroid cancer.

Author (year)/country	Age/sex	Cancer type	Surgery	RAI therapy total dose	Surgery for paranasal sinus mass	Involvement of paranasal sinus metastasis	Other distant metastasis sites	Other treatments	Outcome (follow-up months)
Barrs (1979)/United States (9)	54/F	FTC	NA	RAI therapy	None	Sphenoid sinus extending to the orbit	NA	NA	Died (5 years after metastasis was diagnosed)
Cinberg (1980)/United States (10)	80/F	FTC	Lobectomy => completion thyroidectomy	100 mci	None	Maxillary sinus	Lungs and bones (spine)	Transantral maxillary artery ligation	NA
Chang (1983)/United States (11)	50/F	FTC with PTC	Total thyroidectomy and right radical neck dissection	NA	Lateral rhinotomy, septectomy, ethmoidectomy, and sphenoidectomy	Nasal cavity, nasopharynx, sphenoid sinus extending into the ethmoid sinus, and the sella	Lungs	None	NA
Renner (1984)/United States (12)	61/F	FTC	Total thyroidectomy and limited neck dissection	500 mCi	Rhinotomy	Sphenoid sinus with bony erosion	Neck and bones (hip)	EBRT	NA
Yamasoba (1994)/Japan (13)	34/F	Occult FTC (0.8 cm)	Total thyroidectomy with paratracheal and modified neck dissection	97.1 mCi	Embolization for a hypervascular mass and surgical mass excision	Maxillary sinus, sphenoid sinus, ethmoid sinus, orbit, the infratemporal fossa, and intracranial cavity	Bones (spine)	Bony metastasis removal (spine)	NA
Cumberworth (1994)/England (14)	74/F	FTC	Total thyroidectomy	RAI therapy	None	Sphenoid, ethmoid, and frontal and maxillary sinuses, along with bowing of the lateral nasal wall	Bones (spine)	None (palliative RT for bone metastasis)	Died (1 week after the disseminated metastatic disease was diagnosed)
Freeman (1996)/Canada (15)	50/M	Multifocal PTCs, differentiation to insular and anaplastic carcinoma	Total thyroidectomy and bilateral neck and mediastinal dissection	120 mCi (at the initial PTC diagnosis), 275 mCi (after the metastasis was diagnosed)	None	Ethmoid and sphenoid sinuses	None	EBRT (50 Gy)	Structural remnant (12 months)
Altman (1997)/United States (16)	81/M	FTC	Completion thyroidectomy	RAI therapy	None	Sphenoid and ethmoid sinuses, extending to the sella and invading the clivus	NA	RT	Died (approximately 1 year after metastasis was diagnosed)
Hefer (1998)/Israel (17)	58/M	FTC	Near total thyroidectomy	RAI therapy	Maxillectomy and en bloc tumor resection	Maxillary sinus	None	Brachytherapy with iridium seeds	Remission (NA)
Bhansali (2003)/India (18)	60/F	Follicular variant PTC	Total thyroidectomy	Planned for RAI	None	Maxillary sinus with intraoral protrusion	Bones (mandible and ribs)	None	Follow-up loss
Argibay (2005)/Spain (19)	53/F	PTC	Total thyroidectomy	800 mCi	Partial resection of the tumor after RAI	Sphenoid and ethmoid sinuses, extending to the hypophyseal gland with the invasion of the optochiasmatic cisternae	None	EBRT (57 Gy)	Persistent (3 years)
Krishnamurthy (2010)/India (20)	31/F	FTC	Near total thyroidectomy	160 mCi	None	Maxillary sinus	None	EBRT (40 Gy)	Remission (4/12 years)
Madronio (2011)/Philippines (21)	53/F	PTC	Completion thyroidectomy with debulking of the sternal mass	Planned for RAI	Debulking of the sphenoid/ethmoid mass	Sphenoid and ethmoid sinuses and inferior frontal lobe (brain)	Cervical LN	None	Persistent (13 months)
Shabestari (2012)/Iran (22)	21/F	MTC	Thyroidectomy => completion thyroidectomy and CND	None	None	Maxillary sinus	NA	None	NA
Krishnamurthy (2013)/India (23)	55/F	FTC	Total thyroidectomy with right functional neck dissection	150 mCi	None	Maxillary sinus with extension to the ethmoid and sphenoid sinuses	Lungs and manubrium sternum	Palliative EBRT (40 Gy)	Persistent (6 months)
Kumar (2013)/India (24)	31/F	FTC	Total thyroidectomy	RAI therapy	None	Maxillary sinus	Legs	None	Remission (7 years)
Fatahzadeh (2015)/United States (25)	43/F	Follicular variant PTC	Total thyroidectomy	RAI therapy	None	Maxillary sinus with destruction of the alveolus posterior to the first molar	Lungs, bones (shoulders and hips), and scalp	Sorafenib therapy	NA
Altinay (2015)/Turkey (26)	68/F	FTC (widely angioinvasive)	Total thyroidectomy	200 mCi	None	Nasal cavity, invading the clivus and sella posteriorly, infiltrating the left cavernous sinus, extending towards the interior of the orbit	Lungs and bones (vertebra and femur)	Radiation to the retro-orbital area	Persistent (NA)
Presented case	48/F	FTC and PDTC	Total thyroidectomy with central LN dissection	150 mCi	Endoscopic sinonasal surgery with right orbital decompression	sphenoid sinus with adjacent bony destruction	Bones (spine, pelvic bone, femur, and tibia) and brain	Lenvatinib and palliative RT	31 months. Died due to cerebral infarction.

FTC, follicular thyroid carcinoma; NA, not applicable; RAI, radioactive iodine; PTC, papillary thyroid carcinoma; EBRT, external beam radiation treatment; RT, radiation therapy; LN, lymph node; MTC, medullary thyroid carcinoma; CND, central neck dissection; PDTC, poorly differentiated thyroid carcinoma.

The median age of the 19 patients with thyroid cancer and paranasal sinus metastasis was 53 years, with three (15.8%) patients being males. Fifteen patients (78.9%) were diagnosed synchronously, while four (21.1%) had paranasal sinus metastasis detected during follow-up after thyroid cancer surgery. Among those with thyroid cancer and concomitant paranasal sinus metastasis, four had a history of thyroid surgery for a thyroid tumor or goiter performed at least 2 years prior. One patient underwent near-total thyroidectomy for multinodular goiter just 1 month before, while our case involved a long-standing thyroid nodule. FTC was the most common type, found in 68.5% of cases, followed by PTC at 31.6%. One patient had FTC and PTC, and our case had FTC and PDTC. Of the six patients with PTC, four had classic PTC, two had the follicular variant, and one had multifocal PTC with insular and anaplastic carcinoma. Sphenoid sinus metastasis occurred in 12 patients (63.2%); among these, five had sphenoid and ethmoid sinus involvement, and three had metastasis to the sphenoid, ethmoid, and maxillary sinuses. Distant metastasis was most frequently observed in the bone (47.4%), followed by the lung (26.3%), with one patient having both lung and bone metastasis. All patients underwent near-total or total thyroidectomy, including completion thyroidectomy, and received or were scheduled for postoperative RAI therapy, except for one patient with medullary thyroid carcinoma (MTC). Additional radiation therapy to the paranasal sinus area was performed in eight patients (42.1%), and palliative radiation therapy for other bone metastases was administered to two patients (10.5%). Tyrosine kinase inhibitors were used in two patients: one patient developed paranasal metastasis during sorafenib therapy, while our case was treated with Lenvatinib after the initial diagnosis. Among the 12 cases with available clinical outcomes, three patients achieved remission, while nine patients had persistent disease, with four confirmed deaths (**Table 2**).

**Table 2:** Clinical characteristics of patients with paranasal sinus metastasis from thyroid cancer.

	n (%)
Age, median, [interquartile range]	53 [34–61] years
Sex, male	3 (15.8)
Synchronous^a^	15 (78.9)
Cancer subtype^b^	
FTC	13 (68.5)
PTC^c^	6 (31.6)
MTC	1 (5.3)
PDTC	1 (5.3)
Paranasal sinus metastasis	
Sphenoid	4 (21.1)
Maxillary	6 (31.6)
Sphenoid and ethmoid	5(26.3)
Sphenoid, ethmoid, and maxillary	3 (15.8)
Unspecified	1 (5.3)
Another distant metastasis^d^	
Lungs	5 (26.3)
Bones	9 (47.4)
Brain	1 (5.3)
Legs	1 (5.3)
None	5 (26.3)
Not applicable	2 (10.5)
Other treatment strategies	
EBRT to the paranasal sinus area	8 (42.1)
Palliative radiation therapy	2 (10.5)
Sorafenib	1 (5.3)
Lenvatinib	1 (5.3)
Outcome	
Remission	3/12
Persistent	9/12
Not applicable	7

FTC, follicular thyroid carcinoma; PTC, papillary thyroid carcinoma; MTC, medullary thyroid carcinoma; PDTC, poorly differentiated thyroid carcinoma; EBRT, external beam radiation treatment.

^a^Four patients previously underwent thyroidectomy due to a thyroid tumor or goiter.

^b^One patient had FTC and PTC.

^c^Two cases of follicular variants and one case of insular and anaplastic carcinoma were included.

^d^Four patients showed lung and bone metastasis.

Symptoms at the time of paranasal sinus metastasis diagnosis and characteristics related to primary thyroid cancer are summarized in **Supplementary Table 1**. Symptoms varied depending on the extent of surrounding tissue involvement, including epistaxis, facial pain, painless swelling, nasal obstruction, check fullness, visual disturbances, headaches, and oral bleeding. Unfortunately, detailed ultrasound findings for primary thyroid cancer with paranasal sinus metastasis are not available in the literature. Of the five patients with preoperative FNA data for thyroid cancer, four were diagnosed with follicular neoplasm, and one result was non-diagnostic. Among the seven reported cases of metastatic paranasal sinus tumors with available immunostaining results, six cases of DTC were positive for thyroglobulin or TTF-1, while one case of MTC exhibited positive staining for calcitonin and TTF-1.

## Discussion

Approximately 80% of patients are diagnosed with thyroid cancer and paranasal sinus metastasis simultaneously. Most patients present with symptoms caused by metastatic lesions, which prompt evaluation of a paranasal sinus mass. Similarly, in cases of nasal cavity metastasis from thyroid cancer, nasal-related symptoms such as nasal obstruction or epistaxis are the main symptoms (27, 28). In these cases, biopsy results often reveal lesions resembling thyroid follicular cells, and immunohistochemical staining raises suspicion of a thyroid origin, leading to further thyroid examination. This process might result in a diagnostic delay; thus, a thorough histological evaluation assessment and careful review of the patient’s thyroid history are essential to facilitate timely diagnosis.

This patient had a long-standing thyroid nodule, but appropriate follow-up was not performed. The thyroid nodule, initially diagnosed as a benign follicular nodule 11 years ago, demonstrated size increase in a follow-up examination 3 years later; however, repeat FNA was not performed at that time. Subsequent FNA conducted at our hospital, following the onset of sphenoid sinus metastasis, indicated a follicular neoplasm. The risk of malignancy varies among thyroid nodules with indeterminate cytologic results, making the decision for diagnostic surgery challenging, especially for Bethesda class IV nodules (follicular neoplasm) (29). A recent meta-analysis indicated that malignancy risk increases with tumor size; however, the risk remains significant across all sizes for Bethesda class IV nodules (30). A follicular neoplasm is differentiated from benign thyroid nodules based on cytoarchitectural features observed in FNA (31). Thyroid nodules with any suspicious US features require follow-up US and repeat FNA, particularly if there is an increase in size, to account for the possibility of false negatives.

Nearly all patients undergo thyroidectomy and RAI therapy, with additional radiation therapy often performed in the paranasal sinus area. Depending on the remission status, systemic treatments such as tyrosine kinase inhibitors might be administered, and palliative radiation therapy might be used to address bone metastases. Additionally, a multidisciplinary approach is crucial for managing associated symptoms, including pain management through nerve blocks, local treatments, and addressing visual disturbances, as well as controlling nasal or oral bleeding.

The prognosis for DTC or PDTC with distant metastasis is generally poor, and outcomes for metastasis to rare sites (1, 32), such as paranasal sinus metastasis, remain unclear due to limited data. In our case, stable disease was achieved for 29 months with Lenvatinib therapy, supported by a multidisciplinary team. This case highlights that even with uncommon metastasis locations, a multidisciplinary approach that includes targeted therapies, such as tyrosine kinase inhibitors (6, 33), and collaborative management tailored to the anatomical complexity of the lesion, might improve therapeutic effectiveness.
