# Supplementary figures and images for: Challenging diagnosis and treatment of rare paranasal sinus metastasis from thyroid cancer: a case report and literature review
Source: Front Endocrinol (Lausanne). 2025 May 5;16:1550831. doi: 10.3389/fendo.2025.1550831 (PMC12086075; doi:10.3389/fendo.2025.1550831)

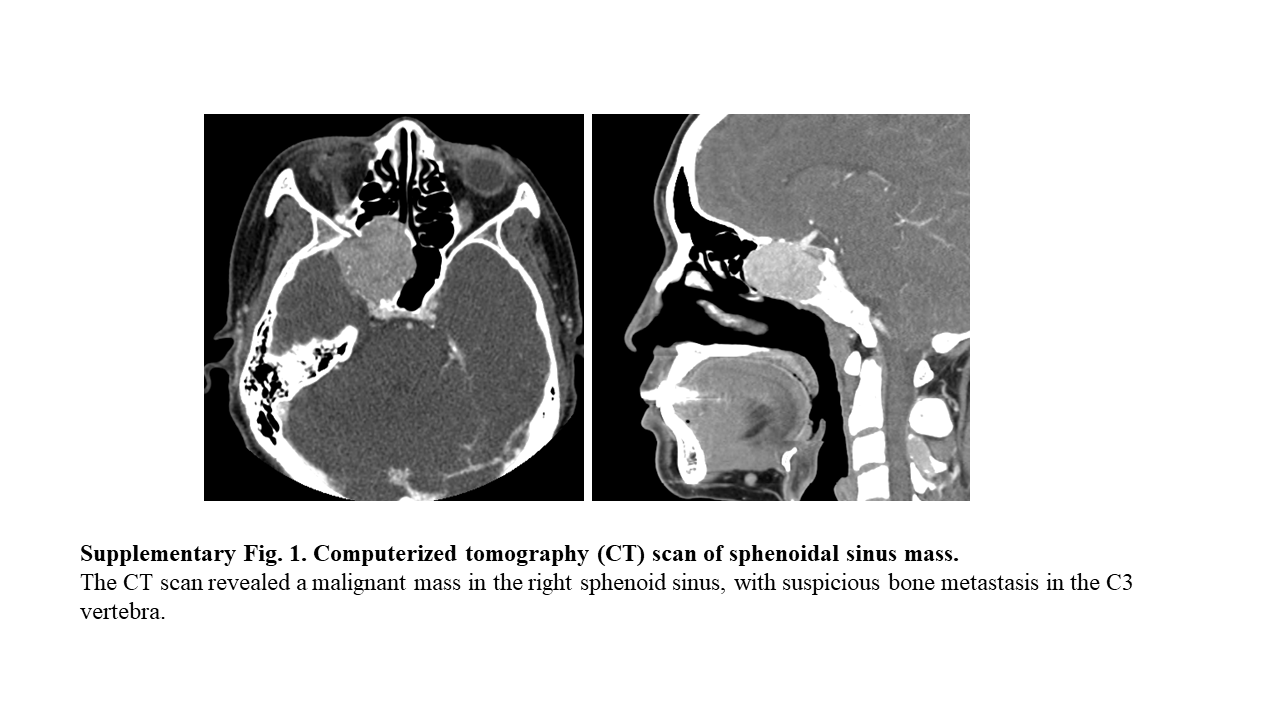

Supplement: Supplementary file 1 [file Image1.tif]

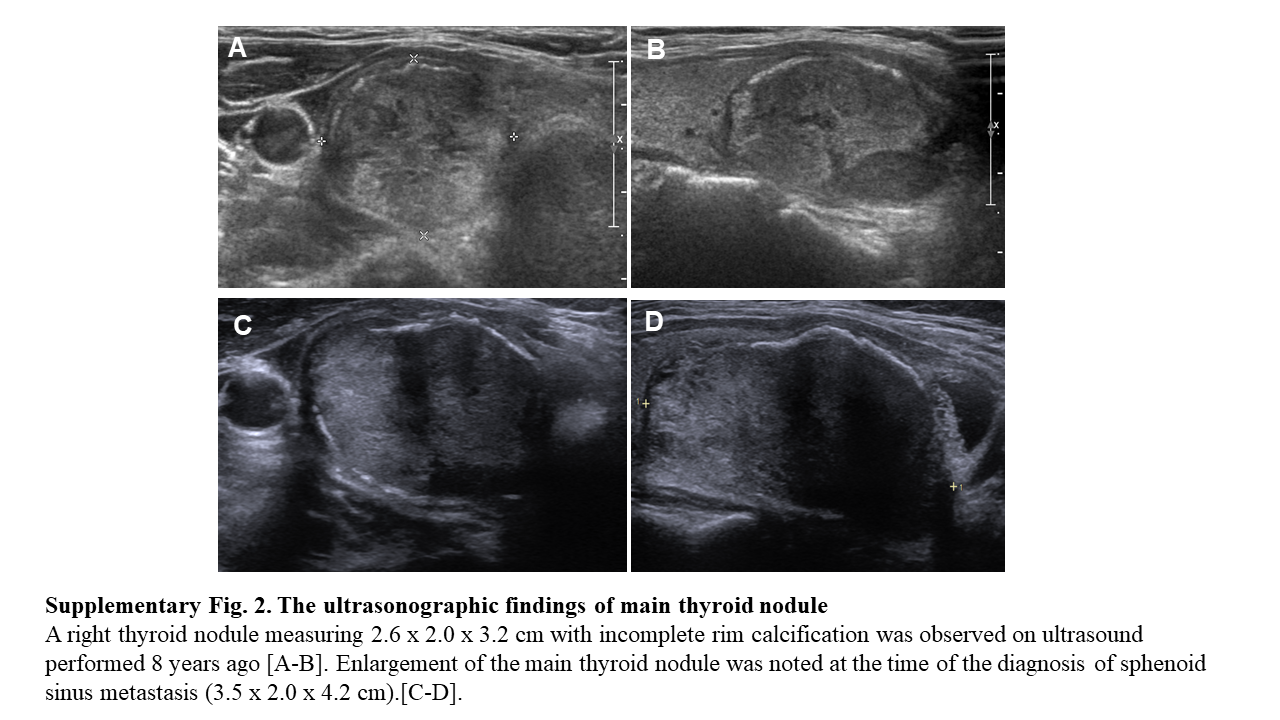

Supplement: Supplementary file 2 [file Image2.tif]

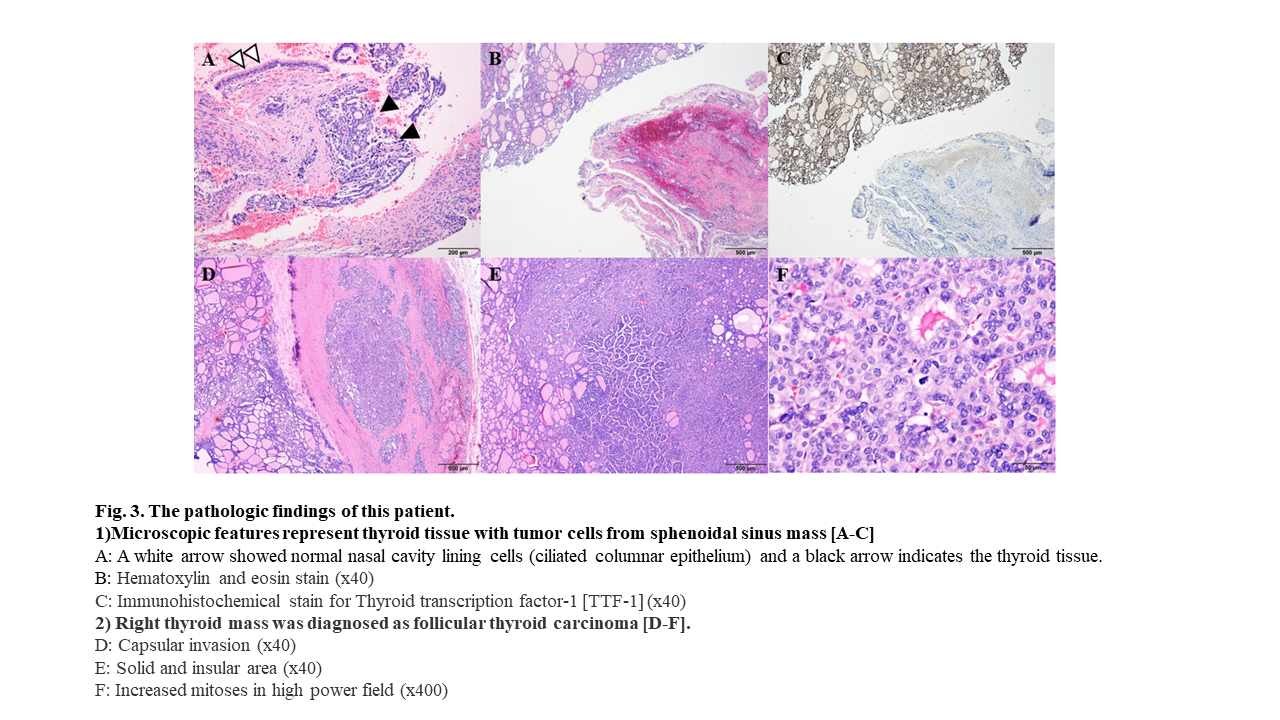

Supplement: Supplementary file 3 [file Image3.tif]

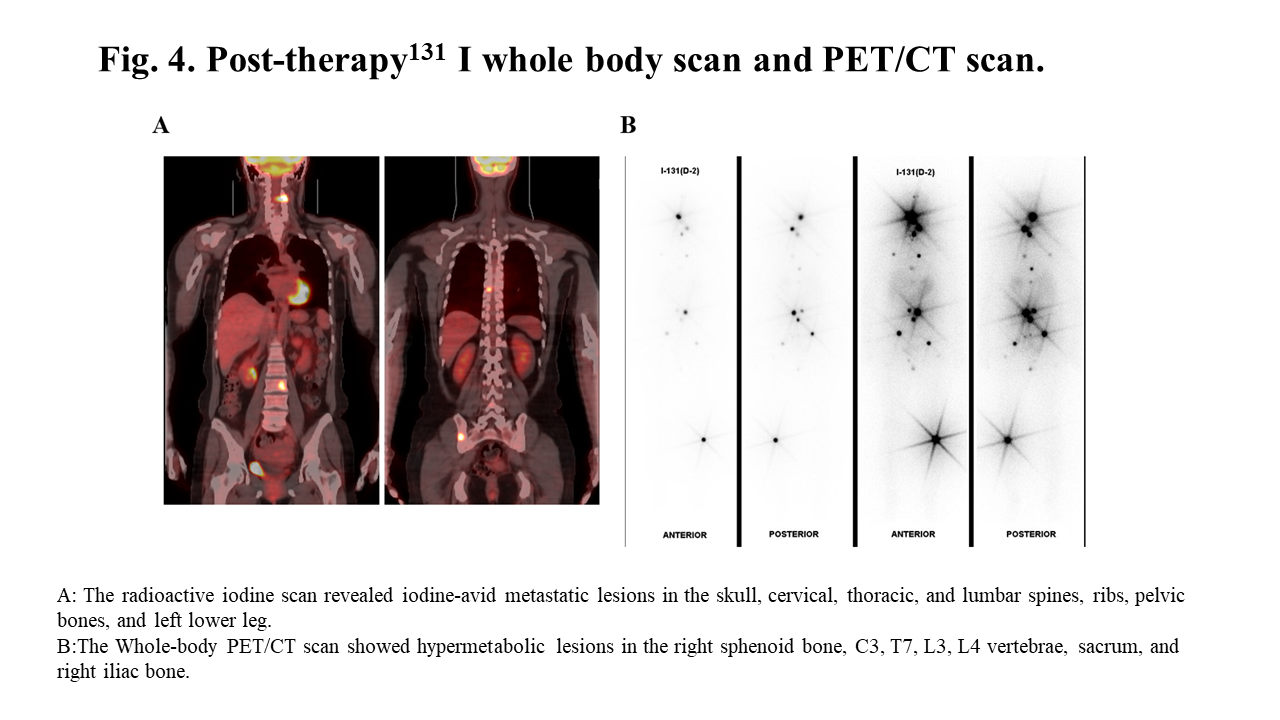

Supplement: Supplementary file 4 [file Image4.tif]

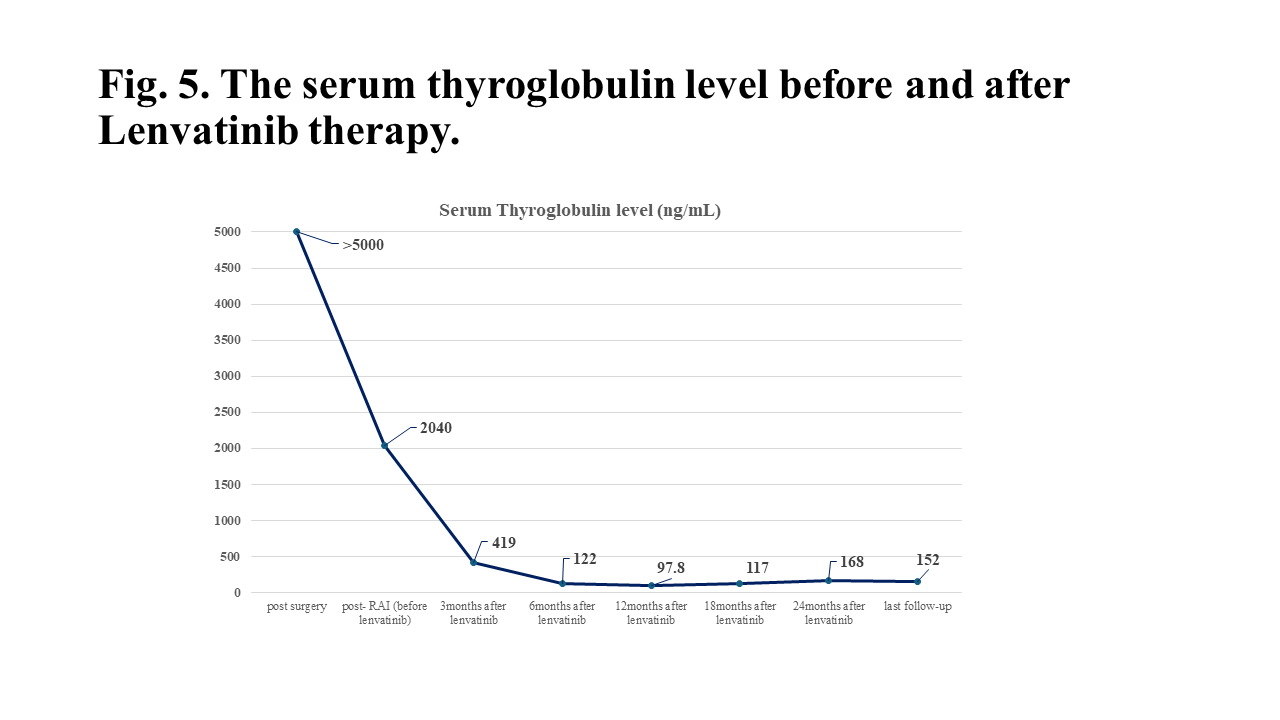

Supplement: Supplementary file 5 [file Image5.tif]
