# Supplementary material for: Challenging diagnosis and treatment of rare paranasal sinus metastasis from thyroid cancer: a case report and literature review
Source: Front Endocrinol (Lausanne). 2025 May 5;16:1550831. doi: 10.3389/fendo.2025.1550831 (PMC12086075; doi:10.3389/fendo.2025.1550831)
Supplement: Supplementary file 6 [file Table1.docx]

**Supplementary Table 1.     Symptoms at the time of diagnosis of paranasal sinus metastasis and thyroid-related characteristics of reported cases.**

| Author (year)/country | Age/Sex | Previous thyroid disease | Symptom | Thyroid US | Main tumor size on US | FNA | Immunohistochemical stain of para-nasal metastatic tumor |  |
| --- | --- | --- | --- | --- | --- | --- | --- | --- |
| Barrs (1979) /USA [9] | 54/F | Simultaneous | Progressive visual loss | NA | NA | NA | NA |  |
| Cinberg (1980) /USA [10] | 80/F | Thyroid tumor removal (16 years ago) | Massive epistaxis | NA | NA | NA | NA |  |
| Chang (1983)/USA [11] | 50/F | Simultaneous | Intermittent epistaxis, weight loss, nasopharyngeal area pain | NA | NA | NA | NA |  |
| Renner (1984)/USA [12] | 61/F | Total thyroidectomy d/t FTC (3 years ago) and RAI therapy for bone metastasis (1year ago) | Epistaxis | NA | NA | NA | NA |  |
| Yamasoba (1994)/ Japan [13] | | 34/F | Simultaneous | Hearing loss, hyperesthesia in the cheek | NA | NA | NA | NA |
| Cumberworth (1994)/ England [14] | 74/F | Total thyroidectomy d/t FTC (12 years ago) | Nasal obstruction | NA | NA | NA | NA |  |
| Freeman (1996)/ Canada [15] | 50/M | Simultaneous  3months after initial diagnosis | Rapid enlargement of neck mass (at the initial diagnosis of  =>facial pain | NA | NA | NA | P53 |  |
| Altman (1997) /USA [16] | 81/M | Partial thyroidectomy d/t multinodular goiter (12 years ago) | Progressive, severe intermittent headaches | NA | NA | NA | Tg (+) |  |
| Hefer (1998)/ Israel [17] | 58/M | Near total thyroidectomy d/t FTC (6months ago) | Hard palate discomfort | NA | NA | NA | Tg(+) |  |
| Bhansali (2003) /India [18] | 60/F | Simultaneous | Painless Cheek swelling | Na | NA | Follicular neoplasm | Tg (+) |  |
| Argibay (2005) /Spain [19] | 53/F | Simultaneous,  Detected on post-op RAI scan after PTC surgery. | Headache, paresthesia in the ciliaris region, eyelid, and right eye, occasional diplopia | NA | NA | NA | NA |  |
| Krishnamurthy (2010)/India [20] | 31/F | Near total thyroidectomy d/t multinodular goiter (1 month ago) | Fullness in the region of the maxillary sinus | NA | NA | NA | NA |  |
| Madronio (2011)/Philippines [21] | 53/F | Left thyroidectomy d/t growing neck mass (2 years ago) | Blurred vision | Recurred solid mass in the left inferior thyroid bed | 6.5cm | Non-diagnostic | NA |  |
| Shabestari (2012)/ Iran [22] | 21/F | Thyroidectomy d/t goiter (7 years ago) | Expansile painless swelling in the maxilla, back pain, dysphagia, chills | NA | NA | NA | Calcitonin (+), TTF-1 (+), Tg (-) |  |
| Krishnamurthy (2013)/India [23] | 55/F | FTC (2 years ago) | Facial swelling, nasal obstruction, occasional epistaxis | NA | NA | NA | NA |  |
| Kumar (2013)/India [24] | 31/F | Simultaneous | Mobile teeth and maxilla swelling | Multinodular goiter | NA | Follicular neoplasm | NA |  |
| Fatahzadeh (2015)/ USA [25] | 43/F | Sorafenib therapy due to Follicular variant PTC | Traumatic ulceration and bleeding during oral functions | NA | NA | NA | Tg(+) |  |
| Altinay (2015)/ Turkey [26] | 68/F | Simultaneous | Puffiness in the eye, vision loss, facial numbness, proptosis | Multinodular goiter | Nearly 5cm | Follicular neoplasm | TTF-1(+), Tg (+) |  |
| Presented case | 48/F | Simultaneous | right visual disturbance and partial nasal obstruction | Multinodular goiter | 4.2cm | Follicular neoplasm | TTF-1(+), PAX8(+) |  |

CND; central neck dissection, PTC; papillary thyroid carcinoma, FTC; follicular thyroid carcinoma, NA; non applicable.
